# Temporal trend in overweight among adolescents in northeastern Brazil

**DOI:** 10.1590/2359-3997000000123

**Published:** 2016-01-01

**Authors:** José Cazuza de Farias, Gerfeson Mendonça

**Affiliations:** 1 Universidade de Pernambuco Programa Associado de Pós-graduação em Educação Física, Universidade de Pernambuco (UPE),; Universidade Federal da ParaíbaJoão PessoaPBBrasil Universidade Federal da Paraíba (UFPB), João Pessoa, PB, Brasil; 2 Universidade Federal da ParaíbaJoão PessoaPBBrasil Universidade Federal da Paraíba (UFPB), João Pessoa, PB, Brasil; 3 João PessoaPBBrasil Grupo de Estudos e Pesquisas em Epidemiologia da Atividade Física (GEPEAF), João Pessoa, PB, Brasil

**Keywords:** Adolescents, overweight, nutritional status, sociodemographic factors, trend

## Abstract

**Objective:**

This study aimed to determine the prevalence and the temporal trend in overweight, as well as their relationship with sociodemographic factors in adolescents from northeastern Brazil.

**Subjects and methods:**

We analyzed data from two similar school-based, cross-sectional epidemiological studies conducted in 2005 (n = 2,768) and 2009 (n = 2,776), with public and private high school students of both sexes, aged 14 to 18 years, in João Pessoa, state of Paraiba, Brazil. Sociodemographic information (sex, age and economic class), body weight (kg) and height (cm) were self-reported by the students. Body mass index was classified according to International Obesity Task Force criteria.

**Results:**

There was a significant increase of three percentage points in overweight prevalence from 2005 (10.1%; CI95%: 8.9-11.3) to 2009 (13.1%; CI95%: 11.4-15.5), which was of greater magnitude in males (5.0%; p < 0.001) than in females (1.8%; p = 0.085). Adjusted analysis showed that the Odds Ratio for an adolescent to show overweight in 2009 compared with 2005 was 1.34 (CI95%: 1.13-1.60), which was also greater in males (OR = 1.45; CI95%: 1.15-1.83), in adolescents aged 16 years (females – OR = 3.01; CI95%: 1.62-5.55), and in those from the middle economic class (males: OR = 1.47; CI95%: 1.03-2.23; females: OR = 1.59; CI95%: 1.01-2.53).

**Conclusions:**

Prevalence of overweight in adolescents was high, and showed an increasing trend, particularly in males who belonged to the middle economic class.

## INTRODUCTION

Prevalence of overweight has been shown to be high and increasing in different population groups, including young people (children and adolescents) (1,2). Overweight adolescents are more susceptible to different health problems, such as arterial hypertension, dyslipidemias, type 2 diabetes metabolic syndrome, depression, low self-esteem, low academic achievement, and lower quality of life (3). Besides, these adolescents are at risk for becoming overweight adults with greater chances of morbimortality by cardiovascular diseases, type 2 diabetes and some types of cancer (1,3). In 2010, it was estimated that overweight and obesity were responsible for 3.4 million deaths all over the world, 4% years of potential life lost and 4% disability-adjusted life years (4), being characterized as one of the major problems in public health (1). Therefore, monitoring of overweight and obesity has been recommended (1).

Although higher prevalence in overweight has been historically identified in adolescents in developed countries, mainly in North America and Europe, in the last decades this scenario started to be observed in Central and South America, and in some countries in Asia and Africa (2). In Brazil, data on the Household Budget Survey of 2008-2009 (POF) (5) demonstrated that overweight prevalence was 21.5% in adolescents from 10 to 19 years of age, and from 14.7% to 17.7% in those between 14 to 19 years old. These findings reinforce the supposition that excess body weigh in not an exclusive problem of developed countries, affecting adolescents in countries at different levels of economic development (2).

The overweight prevalence substantially increased in the last three to four decades. The analysis of data from adolescents in 183 countries demonstrated an increase prevalence of this outcome in all continents, varying with the level of economic development of the country and/or region (2). In the last 10 years, in developed countries, the prevalence of overweight has become stable or slightly reduced, whereas in developing countries it still shows a gradual growth (2).

In Brazil, after 34 years of the first national survey on adolescent nutritional status from 10 to 19 years of age (ENDEF – National Survey of Family Expenditure, 1974-75), the prevalence of overweight increased six times in males (from 3.7% to 21.7%), and almost three times in females (from 7.6% to 19.4%) (5). However, little is known on the temporal trend of overweight prevalence and its behavior in young population of different sociodemographic strata in northeastern Brazilian capitals (6). Brazil underwent deep social and economic changes (increases in gross domestic product, monthly income, purchasing power for minimum wage earnings, greater access to credit, income distribution and welfare programs), and the northeastern region presents the greatest increase in these indicators.

As there is a socioeconomic gradient in overweight (7-9), it is possible that its temporal distribution and trend in sociodemographic strata vary with the socioeconomic condition of each country, or in regions of the same country.

In developed countries, there is an inverse relationship between socioeconomic indicators and overweight (7,8), whereas in developing countries, this relationship is positive (10,11) or has no defined pattern (5,12,13). This finding reinforces the need for studies with this approach in the northeast region of the country. Therefore, the present study described the prevalence and temporal trend in overweight in a four-year period, as well as their association with sociodemographic factors in adolescents of northeastern Brazil.

## SUBJECTS AND METHODS

The study analyzed two similar school-based, cross-sectional epidemiological surveys, that were carried out in 2005 and 2009, with representative samples of high school students from private and public schools in the city of João Pessoa (PB), northeastern Brazil. The projects were approved by the Research Ethics Committee from Universidade Federal da Paraíba (2005 study – 52^nd^ Committee meeting from June, 22^nd^, 2004; 2009 study – protocol number 0062/2009); parents/guardians authorized the participation of all adolescents < 18 years of age to take part in the study.

In both surveys, the sample was selected by cluster in two stages. In the first stage, high school were systematically chosen and proportionally distributed according to size (number of enrolled students), type (public or private school), and demographic region of the city (north, south, east, west). In the second stage, classes were randomly selected for the study and distributed proportionally by shift (morning or evening), and grade (10^th^, 11^th^, or 12^th^ grade).

Data collection in 2005 was carried out between March and October, and in 2009 from May to September. Both collections were carried out by a trained team composed students from the Physical Education course. The teams took part in pilot studies that had the same conditions of the main studies, and followed a data collection protocol described in a manual. An anonymous questionnaire was filled up by the adolescents themselves, in their classrooms, at their regular class times, according to previous instructions provided by the data collection team.

Sociodemographic variables analyzed in the study were: sex (male and female), age (determined by the difference between birth date and date of data collection) and economic classe (using the ABEP (14) [Brazilian Association of Research Companies] methodology, that takes into account the number of material goods and monthly-paid employees in the household, the educational level of the main breadwinner, and clustering in economic classes A1/A2, B1/B2, C1/C2, D and E, which are later on regrouped in: A/B [better condition], C and D/E [worse condition]).

Nutritional status was analyzed by the body mass index (BMI – body weight [kg]/height [m]^2^), based in measures self-reported by the adolescents. The nutritional status classification was based on the criterion of the International Obesity Task Force (15). Adolescents were classified in “not overweight” (low weight + normal weight) or “overweight” (overweight + obesity).

Descriptive analyses included calculations of the proportions and 95% confidence intervals for the categorical variables, and means and standard deviations for numerical variables. The Chi-square test was used to compare the proportion of overweight students between the categories of sociodemographic variables for the data in the 2009 study, and between the 2005 and 2009 studies. The independent t test was used to compare mean BMI scores of the adolescents between 2005 and 2009, according to sex.

Unadjusted and adjusted binary logistic regression were used to evaluate the association between overweight and the sociodemographic variables in the 2009 sample, and the possible changes in the prevalence of this outcome from 2005 to 2009, adjusted by these variables. Overweight (no = 0 and yes = 1) was used as a dependent variable and the year of the survey as the independent variable (2005 = 1 and 2009 = 2). The analyses were stratified by sex and repeated for each subgroup (age: 14, 15, 16, 17, 18 years old; economic classe: A/B, C, D/E), and respectively adjusted by age and economic classe. Odds ratio equal to 1.0 reflected a stable chance of overweight in the period of analysis; values between 0 and 0.99 indicate decreasing chances over time, with the greatest decrease with ratios closer to 0; results over 1.0 reflected growing chances; the greater the magnitude of the increase in the period of analysis, the greater the odds ratio.

All statistical procedures were carried out in Stata 12.0 and took into account the procedure followed for sample selection (cluster sample), using the *svy* option in this software. The analyses were stratified according to sex and economic classe (16). Two-tailed analyses were used in all cases and significance level was set at 5%.

## RESULTS

The sample studied in 2005 was made up by 2,768 adolescents (7.6% losses and refusals) and in 2009, by 2,776 adolescents (20.2% losses and refusals). There were no significant changes in the proportion of adolescents according to sex, age, and mean age between the samples of 2005 and 2009, except for economic classe, which showed an increase in the proportion of middle class adolescents (class C), followed by a reduction in lower classes (classes D/E) – Table 1. For the body mass index, there were significant increases in the mean values for adolescents of both sexes between 2005 and 2009 (females: 20.5 ± 3.2 *vs.* 21.1 ± 2.8: t = -3.50, p < 0.001; and males: 20.8 ± 3.1 *vs.* 21.3 ± 3.5: t = -3.28, p < 0.001) – data not shown in the Tables.

**Table 1:** Characteristics of the sample in the 2005 and 2009 study in adolescents from João Pessoa, Paraíba state (PB), Northeastern Brazil

Variable	2005 study			2009 study		P value
	2009 study	%		n	%	
Total	2,768	-		2,776	-	-
Sex						
Male	1,222	44.5		1,163	42.1	0.128
Female	1,546	55.5		1,598	57.9	
Age (years)						
14	297	10.9		356	12.8	0.056
15	622	22.8		772	27.8	
16	814	29.9		823	29.6	
17	637	23.4		615	22.2	
18	355	13.0		210	7.6	
Economic classes						
A,B	866	37.8		997	40.6	< 0.001
C	926	40.4		1,124	45.8	
D,E	499	21.8		333	13.6	

**Figure 1:**
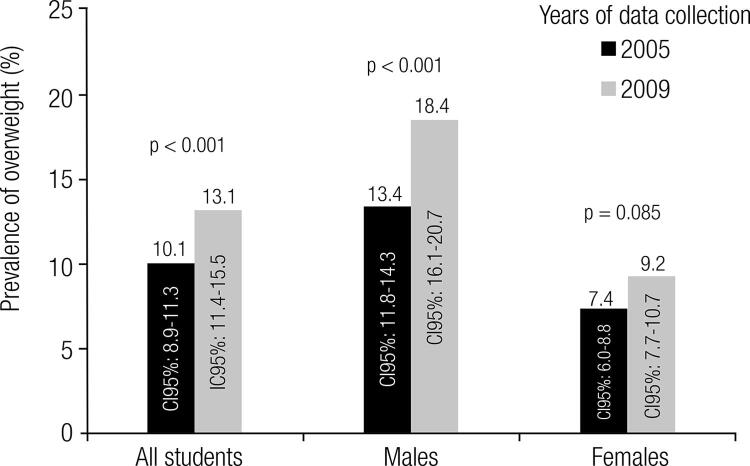
Prevalence of overweight in adolescents from João Pessoa, Paraíba state (PB), Northeastern Brazil in 2005 and 2009.

The prevalence of overweight in 2009 was 13.1% (CI95%: 11.1-15.5), greater in males (18.4%; CI95%: 16.1-20.7) than in females (9.2%; CI95%: 7.7-10.7; p < 0.001) – Figure 1. Table 2 shows the prevalence of overweight in 2009 by sociodemographic factor categories, stratified by sex. In males there was a positive and significant association between overweight and economic classe, indicating that those 16 years of age (OR = 1.95, CI95%: 1.04-3.65) and those that belonged to A, B economic classes (OR = 2.70, CI95%: 1.12-6.50) had greater chances of being exposed to overweight.

**Table 2:** Prevalence of overweight and association of sociodemographic factors in adolescents from João Pessoa, Paraíba state (PB), Northeastern Brazil, 2009 (n = 2,768)

Variable	Male (n = 1,222)					Female (n = 1,546)			
	n	%	P value	Odds Ratio (CI95%)***		n	%	P value	Odds Ratio (CI95%)***
Age (years)			0.735**					0.337*	
14	17	14.3		1		24	11.7		1
15	58	19.5		1.83 (0.97-3.47)		32	7.6		0.58 (0.32-1.06)
16	69	21.5		1.95 (1.00-3.65)		50	11.4		0.98 (0.57-1.70)
17	42	16.1		1.32 (0.68-2.56)		22	6.8		0.55 (0.29-1.05)
18	15	15.6		1.24 (0.53-2.53)		9	8.7		0.46 (0.17-1.28)
Economic classes			0.009*					0.097*	
A,B	109	22.0		2.70 (1.12-6.50)		44	7.6		0.59 (0.30-1.13)
C	71	17.5		2.05 (0.84-4.97)		61	9.5		0.75 (0.39-1.42)
D,E	6	9.5		1		13	11.8		1

* Chi-square test for linear trend; ** Chi-square test for heterogeneity; *** Adjusted by age and economic classe.

As for the temporal trend in overweight prevalence, a significant increase of three percentage points was observed from 2005 (10.1%) to 2009 (13.1%). This increase was greater in males (increase in 5.0 percentage points) than in females (increase in 1.8 percentage points) – Figure 1.

Table 3 shows the results of the comparison between the prevalence of overweight between 2005 and 2009, with all participants of the study, as a function of age and economic classe, for the analysis stratified by sex. After adjusting by age and economic classe, it was observed that the Odds Ratio for an adolescent to present overweight in 2009 compared with 2005 was 1.34 (CI95%: 1.13-1.60) in the analysis of all participants in the study, and 1.45 (CI95%: 1.15-1.83) in males, but with no statistical significance in females, 1.27 (CI95%: 0.97-1.66) – data not shown in the Tables. For both sexes, the chance of overweight in the 2009 study, compared with 2005, was greater in those who were 16 years of age (females: OR = 3.01; CI95%: 1.62-5.55) and in the middle class (males: OR = 1.47; CI95%: 1.03-2.23; females: OR = 1.59; CI95%: 1.01-2.53) – Table 3.

**Table 3:** Comparison of the prevalence of overweight between 2005 and 2009 in adolescents from João Pessoa, Paraíba state (PB), Northeastern Brazil

Variable	2005 vs. 2009 study Males							2005 vs. 2009 study Females					
	2005			2009		Odds Ratio (CI95%)*		2005			2009		Odds Ratio (CI95%)*
	n	%		n	%			n	%		n	%	
Age (years)**													
14	15	15.6		17	14.3	0.88 (0.37-2.10)		19	11.9		24	11.7	1.22 (0.57-2.60)
15	38	16.3		58	19.5	1.52 (0.92-2.51)		25	8.4		32	7.6	0.85 (0.45-1.62)
16	43	13.9		69	21.5	1.46 (0.94-2.28)		25	6.1		50	11.4	3.01 (1.62-5.55)
17	30	10.5		42	16.1	1.69 (0.96-2.98)		16	5.5		22	6.8	1.08 (0.53-2.20)
18	17	12.2		15	15.6	1.03 (0.43-2.43)		14	8.0		9	8.7	0.81 (0.26-2.49)
Economic classes***													
A,B	61	17.7		109	22.0	1.34 (0.94-1.90)		21	7.2		44	7.5	0.98 (0.57-1.70)
C	41	12.3		71	17.5	1.47 (1.03-2.23)		29	6.2		61	9.5	1.59 (1.01-2.53)
D,E	16	7.8		6	9.5	1.46 (0.53-3.99)		25	7.8		13	11.8	1.58 (0.77-3.21)

* Results of the logistic regression were adjusted by economic classe and age. The reference category was the presence of overweight/obesity in 2005.

** Adjusted by economic classe.

*** Adjusted by age.

## DISCUSSION

The present study described the prevalence and temporal trend in overweight and analyzed their association with sociodemographic factors in adolescents of northeastern Brazil. The proportion of overweight adolescents was relatively high and presented a significant increase from 2005 to 2009, mainly in males of lower economic classes.

The prevalence of overweigh in the present study for 2009 (13.1%) was lower than in international studies [USA: 33.6% (16); China: 19.1% to 33.2% (17); Australia: 27% (18); European countries: 14% to 28% (19)] and in the Household Budget Survey (POF 2008-2009) (5) for adolescents between 10 to 19 years of age in almost all regions of the country (North = 18.5%, Center-West = 23.9%, Southeast = 24.4%, South = 26.9%), and was closer to that of the Northeast region (15.9%). Studies conducted in adolescents in Northeastern Brazil [Maceió, AL = 13.8% (20); Pernambuco = 13.9% (21)] identified prevalence similar to that of the present study. Overall, these findings confirm the presence of lower overweight prevalence in regions of less socioeconomically developed regions.

As observed in other studies conducted in adolescents in Europe (19), USA (16) and different regions of Brazil (5,22,23), in the present study the prevalence of overweight was greater in males. However, some studies show higher prevalence for females (12) or no differences between the sexes (21). Greater overweight prevalence in males may be explained by the lower sensitivity of cutoff points that define excess weight based on BMI in females (24), greater consumption of hypercaloric food and longer periods of sedentary behavior, and lesser concern of male adolescents with the repercussions of overweight and body aesthetics (9,25). On their turn, females have greater concern of remaining thin as they may want to comply with the “beauty and body aesthetics patterns” (26).

In males, greater exposure to overweight occurred at 16 years of age in higher economic classes. These results are different from those observed in adolescents in developed countries (2,7,8) and similar to those reported in developing countries (11,13,17). In Brazil, systematic reviews (6,12) identified a positive association between socioeconomic indicators (education level, economic classe, monthly income) and overweight in males, and an inverse or absent association in females (6,12). This association pattern is typical of countries undergoing nutritional transition, which is characterized by an inversion in the distribution pattern of nutritional problems, marked by the passage for undernutrition to overweight and obesity as one of the major public health problems, in response to modifications in diet, physical activity, socioeconomic and demographic conditions (9).

In developing countries, improved socioeconomic conditions lead to the consumption of industrialized, ultra-processed foods, ready-to-eat meals, soft drinks, saturated and trans fat, that are frequently observed in higher socioeconomic strata, which may explain the greater occurrence of overweight among adolescents from richer families (27).

The analysis of temporal trends in overweight showed a significant increase of three percentage points from 2005 to 2009 in adolescents in João Pessoa (PB), representing a mean increase of one percentage point per year (0.75 percentage point). This increase was greater among males and in the middle economic classe in both sexes. These results are similar to studies in developing countries (11,13,17), including Brazil (5), which still shows an increase in the prevalence of overweight among adolescents, different from developed countries. In their majority, developed countries show an stabilization (18,28) or slight reduction (16,19,29) in this trend.

A study of the temporal trend by the Swiss government (29) demonstrated that from 2002 to 2007, overweight in male and female adolescents was significantly reduced, in average by 3.1 and 5.6 percentage points in the period, corresponding from 0.62 to 1.12 percentage points a year, respectively. Similar results were observed in adolescents in France (28) and Australia (18). In the US, data from the last surveys [2007-2008 and 2009-2010 (16)] from the National Health and Nutrition Examination Survey – NHANES – demonstrated a reduction in the prevalence of overweight, from 34.2% to 33.6% (average reduction of 0.3 percentage point a year). A study published in the Lancet showed that in developed countries, overweight prevalence is decelerating or stabilizing in the last 10 years, whereas it is still increasing in developing countries (2). These results indicate that developed countries are moving towards the end of the nutritional transition process, whereas it is still evolving in developing countries (9).

In Brazil, it seems that the end of nutritional transition is not close, as the proportion of overweight adolescents is still growing. Results of the last POFs [2002-2003 (30) and 2008-2009 (5)] demonstrate that overweight among adolescents increased in about seven points in males (0.83 percentage points/year), and five points in females (0.71 percentage points/year). These increases were lower than those of the present study (males = 1.25 percentage points/year and females = 0.45 percentage points/year).

Some factors may explain the increased overweight prevalence among adolescents, particularly in relation to physical activity, sedentarism and feeding habits. The decrease in the number of spaces destined to physical activities, increased urban violence and use of cars in the cities have contributed for the decreased level of activity among adolescents (9). In parallel, adolescents have use growing time in sedentary activities (watching TV, using computers, playing videogames, using cell phones) (25).

Changes in the feeding habits and behaviors among Brazilians in the last three to four decades have also contributed with this scenario. The analysis of population surveys on feeding patterns among Brazilians showed a growing number of meals outside the house (from 24% of total food expenses in 2002-2003 to 34% in 2008-2009) (31), consumption of industrialized, ultra-processed foods and substitution of meals and traditional preparation for quick snacks (27). Industrialized, ultra-processed foods consumed outside the households and snacks are high calorie-dense foods, with high total fat, saturated and trans fats, simple carbohydrates and sodium, and low fiber concentration. Adolescents show the highest rate of consumption of foods outside the house and greater average consumption of cakes and cookies, industrialized salty snacks, milk and dairy products, sweets, soft drinks, juice, fried foods and sandwiches (32). These foods are among the ten more consumed by adolescents in Brazil (33). Another important change was the increased consumption of fast food and the increased size of the portions (9).

An important result identified in the present study was greater overweight prevalence in adolescents of both sexes in the middle class in the period analyzed (2005-2009). These findings suggest that overweight distribution among adolescents in moving towards a pattern similar to that observed in developed countries, characterized by overweight prevalence in lower socioeconomic strata (2,9). The comparison of data from the 2002-2003 and 2008-2009 POFs revealed greater increase in overweight prevalence in adolescents that belonged to the bottom fifth in income for females, and in the top fifth, for males (5,30). These behaviors are typical of countries undergoing nutritional transition (9).

The increased overweight prevalence in adolescents of the middle class may be due to the increased proportion of families in this economic classe that have higher income, greater purchasing power, and access to credit that were observed in Brazil in the last decade. These factors influenced the habits and food behaviors of the population and are intimately associated with the increased consumption of meals outside the house, consumption of industrialized and ultra-processed foods, soft drinks, snacks, and saturated and trans fat. On the other hand, greater access to information on the risks of overweight, given higher educational levels and access to healthier foods and greater purchasing power, are providing more protection against overweight in people of higher social strata, especially females.

Based on the two cross-sectional surveys using samples representative of high school students and similar methodology, the present study was one of the first in the northeastern region of Brazil to describe the current prevalence (2009) and the temporal trend (2005-2009) in overweight and to analyze the association between this outcome and sociodemographic factors. Another important approach was the use representative samples of adolescents in high school that had similar characteristics, associated with the low rate of withdrawals and refusals in the two studies, which prevented possible selection bias.

One of the limitations of this study was not including adolescents that were out of school. These adolescents are generally poorer and, therefore, have greater chances of presenting overweight. It is believed that, if they were part of the study, overweight prevalence would be even higher, and the outcomes would be more frequent in poorer females. The use of self-reported weight and height measures was another limitation of the study. Although these measures were in agreement with actual measurements, they self-reported measures tend to underestimate the prevalence of overweight, particularly in females (34), which may have contributed to an underestimation of overweight prevalence in females, mainly in lower economic classes.

Based on the data analyzed here, it may be concluded that overweight prevalence in 2009 was high, mainly in male adolescents of 16 years of age and of higher economic classes. The temporal trend in the prevalence of overweight showed that this important public health problem continues to grow among adolescents in northeastern Brazil at a rate higher than in many developed countries. This increase was greater in male adolescents and in those of lower economic classes, in both sexes. These findings reinforce the need to develop actions directed to the treatment and prevention of overweight among adolescents in northeastern Brazil. Interventions in the school that emphasize the adoption of health feeding habits and an active lifestyle should be developed and articulated with health promotion public policies.
